# Granulocyte-macrophage colony-stimulating factor reduces lung bacterial load following traumatic brain injury and hemorrhage polytrauma in a juvenile rat model

**DOI:** 10.1371/journal.pone.0323674

**Published:** 2025-05-19

**Authors:** Ghaith A. Bahader, Timothy Warner, Mark W. Hall, Eric A. Sribnick

**Affiliations:** 1 Center for Clinical and Translation Research, Abigail Wexner Research Institute at Nationwide Children’s Hospital, Columbus, OH,; 2 Department of Pediatrics, Division of Critical Care, Nationwide Children’s Hospital, Columbus, OH,; 3 Department of Surgery, Division of Neurosurgery, Nationwide Children’s Hospital, Columbus, OH,; 4 Department of Neurosurgery, The Ohio State University, College of Medicine, Columbus, OH; School of Medicine, Tokyo Women's Medical University, JAPAN

## Abstract

Traumatic brain injury (TBI) in children is associated with high rates of morbidity and mortality. Nosocomial respiratory infections are common following severe TBI, especially with polytraumatic TBI. Although post-injury immunosuppression has been identified as a potential contributor to nosocomial infection, the underlying mechanism and optimal therapy are poorly understood. In this study, we used a combined model of TBI plus extra-cranial hemorrhage followed by intranasal inoculation with *Streptococcus pneumoniae* to model the clinical scenario of polytrauma TBI and post-injury infection. Briefly, 28-day (prepubescent) rats received either sham injury or prefrontal controlled cortical impact injury plus removal of 25% of blood volume through femoral cannulation. Saline or 50µg/kg granulocyte-macrophage colony-stimulating factor (GM-CSF) was administered intraperitoneally daily for 2 days. Post-injury immune response was assessed in the blood, spleen, and brain using different immunologic techniques, while bacterial clearance was examined by plating lung tissue. Our results show that GM-CSF enhanced innate immune function by increasing the percentage of blood monocytes expressing elevated levels of MHC II molecules. GM-CSF also significantly increased splenic CD3 + T-cells, compared to the saline-treated injury group. Moreover, increased lung bacterial load (colony-forming units) was significantly reduced with GM-CSF treatment. Treatment with GM-CSF was not associated with an increase in brain glial activation, neuronal loss, or memory dysfunction. This study highlights the potential role of GM-CSF as a therapy to address the increased risk of nosocomial infections following polytraumatic TBI.

## Introduction

Traumatic brain injury (TBI) represents one of the major causes of mortality and long-term morbidity in pediatric patients. In the United States, annual estimates for patients 0–14 years of age include approximately 18,000 hospitalizations and 1,500 deaths related to TBI [1]. The development of post-injury nosocomial infection, in particular hospital-acquired pneumonia and ventilator-associated pneumonia, is a common and major complication of severe TBI [2]. It has been shown that TBI patients have a higher risk for nosocomial infections than non-TBI patients in the intensive care unit, and these infections following severe TBI can increase the mortality rate to as high as 37% [3,4]. Often TBI is accompanied by secondary extracranial injuries, and polytraumatic TBI is associated with even higher risks of morbidity and mortality [5]. We have previously shown that, in children, a TBI plus an extracranial injury is associated with a higher risk of post-injury infection, especially ventilator-associated pneumonia [6].

An important factor that has the potential to impact the rate of post-injury nosocomial infection is the systemic immune response. Following a severe trauma, both the pro-inflammatory systemic immune response and the compensatory anti-inflammatory response have been shown to be activated [7]. In our previous research, we described that reduction of innate immune function in critically injured children is common, particularly in the setting of neurotrauma, and that severe reduction is associated with increased risk for nosocomial infections [6,8]. Furthermore, several preclinical studies have made similar findings in animal models of TBI in which acute post-injury systemic immune suppression was associated with increased pulmonary edema [9] and mortality in response to induced pneumonia infection [10]. We and others have shown that, when compared to TBI alone, a mixed injury model employing systemic hemorrhage and TBI leads to down-regulation of the immune response [11,12]. To investigate the mechanism behind this phenomenon, we previously developed a combined injury model of bilateral frontal brain contusion and systemic hemorrhage in juvenile rats which was shown to consistently cause declines in both innate and adaptive immune function after the injury [12,13]. Our prior research has shown that either component of the injury model alone (i.e., either the hemorrhage or the TBI) does not lead to innate [12] or adaptive [13] immune suppression but that the combination injury does. The combined animal injury model that we use seeks to recreate the scenario of polytraumatic TBI in a preclinical model to allow for testing of the mechanism and treatment of post-traumatic immune suppression.

One way to counteract post-injury systemic immune suppression is the pharmacological stimulation of the immune response. Granulocyte macrophage colony-stimulating factor (GM-CSF), has been shown to play an important role in immunomodulation by affecting the generation, maturation, and differentiation of myeloid cells including monocytes and macrophages [14]. Moreover, it has been demonstrated that GM-CSF can modulate the function of other cell types, including T-cells through GM-CSF stimulation of macrophages with downstream release of IL-23 [15,16]. However, the role of GM-CSF in reversing post-TBI immune suppression and limiting the risk of post-injury nosocomial infection is poorly understood. Additionally, as GM-CSF has been shown to play a role in auto-immune disease [17], there are concerns that its use could enhance neuroinflammation and exacerbate secondary brain injury.

In the present study, we investigated the immunological and neurological effects of post-injury GM-CSF in a combined TBI plus systemic hemorrhage (TBI/H) model with acute post-injury pneumonia induction with *Streptococcus pneumoniae*. Using our TBI/H injury model in juvenile rats, we examined whether treatment with GM-CSF can reverse innate and adaptive immune suppression and enhance the host response to early pneumonia infection. Moreover, we evaluated the impact of GM-CSF treatment on neuroinflammation in the injured brain by measuring the glial cell activation and neuronal loss in the peri-lesional area and by testing spatial memory.

## Materials and methods

### Animal care

All animal protocols were approved by the Institutional Animal Care and Use Committee (IACUC) at Nationwide Children’s Hospital and complied with National Institute of Health (NIH) guidelines. Accordingly, all considerations were taken to minimize suffering and distress, and all staff handling live animals were specifically trained on-site through computer-based learning modules, live training modules, and continuing education by veterinary/research staff. Animals were housed under a 12-hour dark and light cycle with free access to food and water. Animals were monitored by at least daily inspection and weighing. Postoperatively, animals were inspected twice daily. Specifically, animals were monitored for any signs of suffering or distress. Specific humane endpoints for which animals were monitored included loss of more than 15% of body weight in 3 days, lack of response to manual stimulation (handling); immobility; or inability to eat, drink, or groom. In addition, animals were examined for markers of distress including reddened eyes, alopecia or discolored fur, hyper or hypoactivity, or hunched posturing. The total duration of the experiment was 7 days.

A total of 40 juvenile Sprague Dawley male and female rats were used in this study and randomly subdivided into 4 groups: sham injury treated with saline (Sham+saline), sham injury treated with GM-CSF (Sham+GM-CSF), TBI/H injury treated with saline (TBI/H+saline), and TBI/H treated with GM-CSF (TBI/H + GM-CSF). Of the 40 animals used for the outlined experiments, none were excluded for technical reasons, 0 died, 0 showed signs suggesting a humane endpoint had been reached, and all were randomly assigned to treatment groups to maintain a balanced pre-injury weight. All 40 rats received the predetermined therapy, and complete data was obtained. Investigators who administered the therapeutic and performed outcome assessments were blinded to the study groups.

**Fig 1:**
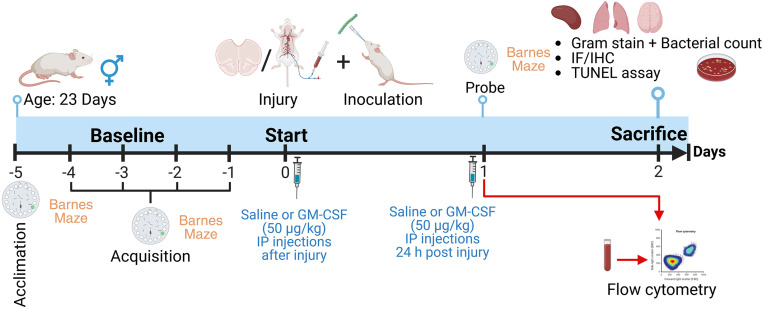
Experimental design overview. Rats were acclimated to the Barnes maze for 1 day (pre-injury day 5) and then the acquisition phase was run for another 4 consecutive days. Surgery to perform Sham or TBI/H injury occurred on day zero, which was post-natal day 28. Surgery was followed by bacterial inoculation. Saline or GM-CSF were administered intraperitoneally immediately after the surgery and on day 1. Blood was collected one day after the surgery for flow cytometry analysis. Rotarod and Barnes maze probe phase were performed on day 1. Rats were sacrificed on day 2, and tissue samples (spleen, brain, and lungs) were collected. Abbreviations include: granulocyte-macrophage colony-stimulating factor (GM-CSF), immunofluorescence (IF), immunohistochemistry (IHC), intraperitoneal (IP), terminal deoxynucleotidyl transferase dUTP nick end labeling (TUNEL).

Rats received TBI/H or sham surgery on day 0 (age post-natal day 28) and were immediately injected intraperitoneally with 50 μg/kg recombinant rat GM-CSF (AF-400–23, PeproTech, Cranbury, NJ) or an equal volume of saline vehicle; respectively. Inoculation with *S. pneumoniae* was also performed on all groups on the day of surgery. The inoculum used was based on prior research and on our own findings regarding the timing of lung bacterial clearance following sham surgery or the TBI/H model (S1 Fig). GM-CSF dosing was repeated on post-injury day (PID) 1, and all rats were sacrificed on PID 2, as outlined in Fig 1. The dose of GM-CSF was selected based on our prior findings that this dose is sufficient to normalize post-injury immune function [18]. For sacrifice, animals were fully anesthetized using a mixture of isoflurane and oxygen and were decapitated using a small animal guillotine. Lungs and spleens were harvested and placed in chilled phosphate-buffered saline (PBS). Brains were collected in 4% paraformaldehyde (PFA, ThermoFisher, Waltham, MA) in PBS.

### Controlled cortical impact (CCI)

All surgical procedures were performed under standard sterile conditions. Rats were anesthetized with isoflurane (5% during induction and 2% during maintenance for the rest of the surgery), and the head was fixed into a stereotaxic frame (Kopf, Tujunga, CA). For the cranial procedure, a longitudinal midline incision was made, and the skin was retracted to expose the skull. Then, a bilateral 5mm craniectomy was performed (centered at 2mm anterior to the bregma), and the skull was removed carefully without damaging the exposed dura. CCI injury was delivered using the Impact One machine (Leica, Deer Park, IL) with a 3mm diameter impactor tip. The following measures were used to deliver the injury: 3mm depth, 500 millisecond dwelling time, and 4 m/s velocity. Immediately following the injury, hemostasis was obtained, and the incision was sutured. Similar procedures were followed for the sham rats including the incision and craniectomy but without the CCI impact.

### Fixed-volume hemorrhage

Immediately following the CCI procedure, a controlled systemic hemorrhage was performed during the same anesthetic session. Animals were removed from the stereotaxic apparatus and repositioned supine. An incision was made over the left inguinal area, and the femoral artery/vein complex was identified. The femoral vein was cannulated with a 27-gauge needle, and 25% of the estimated blood volume (EBV) was withdrawn. EBV was determined based on each rat’s body weight (70 ml/kg) [19]. Following the blood aspiration, the incision was sutured, and rats were allowed to fully recover before returning to their home cage. Sham animals were anesthetized for a comparable duration, and an identical incision and dissection were performed but without any removal of blood.

### Bacterial inoculation

Experimental induction of pneumonia was performed using type 3 *S. pneumoniae* (ATCC 6303, American Type Culture Collection, Manassas, VA). Bacteria were maintained in Brain Heart Infusion Broth (Difco Laboratories, Detroit, MI, USA) and were stored with 20% glycerin at ‐80 °C until used and were resuspended prior to inoculation. Shortly after injury induction and recovery from surgery, rats were anesthetized with 2% isoflurane, and 50 μL of bacteria (5x10^7^ CFUs/mL) suspended in sterile PBS was applied intranasally, allowing the rats to aspirate the liquid.

### Bacterial colony formation measurement

On PID 2, lungs were harvested under sterile conditions (*n* = 7–9 per experimental group), and one lung was mixed with 5 mL of sterile PBS before undergoing homogenization (Qiagen, TissueRuptor II). Subsequently, 200 μL of the homogenized mixture was evenly distributed onto blood agar plates (ThermoFisher) and allowed to incubate overnight at 37 °C. On the next day, colonies were quantified by a treatment-blinded investigator and were reported as colony forming units (CFUs).

### Gram stain

A portion of harvested lung tissue was embedded in paraffin and sliced into 8 µm sections (*n* = 7–9 per experimental group). The Browns and Hopps method for the gram staining was used [20]. Briefly, slides were heated for 10 minutes, deparaffinized, and then stained sequentially with crystal violet stain, iodine, and basic fuchsin. Slides were then differentiated with Gallego solution. Finally, the slides were cleared with xylene and cover slipped with a compatible mounting medium. Images were acquired using a Nikon microscope (Eclipse Ti2, Tokyo).

### Immunohistochemistry

Spleen tissue samples were fixed with paraformaldehyde, embedded with paraffin, and sliced into 8 µm sections (*n =* 5–8 per experimental group). Briefly, slides were deparaffinized and rehydrated with xylene and a graded series of ethanol followed by antigen retrieval with sodium citrate buffer in a pressure cooker for 25 minutes. Slides were then cooled by rinsing in running water and placed in 1X PBS-T for 5 minutes. Endogenous peroxidase activity was quenched by blocking in 3% hydrogen peroxide for 30 minutes at room temperature. Slides were then blocked with a biotin blocking kit (ScyTek Laboratories, Logan, UT) for 30 minutes followed by a superblock solution for another 15 minutes. Spleen tissue sections were incubated overnight at 4°C with rabbit anti-CD3 polyclonal primary antibody (1:100, Abcam) and anti-CD68 (1:100 BioRad), respectively. The next day, slides were washed with PBS-T buffer for 5 minutes, incubated with a secondary anti-polyvalent antibody (ScyTek) for 30 minutes, and incubated for another 20 minutes with horseradish peroxidase with brief rinsing with water between each step. Slides were then developed with 3,3′-diaminobenzidine diluted in distilled water and 1% hydrogen peroxide for 1 minute, counterstained with hematoxylin for 1 minute, followed by bluing solution for 20 seconds, and mounted with mounting media. Images were captured with a Nikon Eclipse Ti2 microscope, and the area of positive cells was quantified using QuPath software by calculating the percentage of chromogen-positive area relative to the white pulp in 5 different randomly chosen areas.

### Flow cytometry

Blood samples (*n* = 8–10 per experimental group) were collected on PID 1 in heparin-coated tubes (BD Biosciences, Franklin Lakes, NJ), and red blood cells were lysed using lysing buffer (BD Biosciences). After blocking the Fc receptor with anti-CD32 antibody (BD Biosciences), cells were washed and incubated for 15 minutes at room temperature with the following antibodies: CD3 (PE, BioLegend, San Diego, CA), CD11 b/c (APC, BioLegend), His48 (FITC, BD Biosciences), major histocompatibility complex (MHC)II RT1B (BV421, BD Biosciences), CD4 (BV786, BD Biosciences). Cells were then washed and resuspended with stain buffer (BD Biosciences). Single color-stained control beads (ThermoFisher, Waltham, MA) were utilized for the compensation experiment. Gating (S2 Fig) was set for forward scatter (FSC) and side scatter (SSC) to discriminate the live cells. Percentage of live cells was used to quantify each cell population and gating was determined based on the proper isotype controls. Data were acquired using LSR II (Becton Dickinson) and analyzed with FlowJo software (Version 10.9.0, BD Biosciences).

### Immunofluorescence

Paraffin-embedded brain and spleen tissues were sliced into 8 µm sections using a microtome and used for staining (*n* = 5–6 per experimental group). Briefly, slides were deparaffinized, and immersed with sodium citrate buffer, followed by antigen retrieval in a pressure cooker for 10 minutes. Afterward, the sections were washed three times with 1 × PBS and blocked with 5% BSA at room temperature for 2 hours. Subsequently, different primary antibodies including rabbit anti-Iba-1 (1:500, Wako), rabbit anti-GFAP (1:500, Abcam), mouse anti-NeuN (1:500, Sigma), and mouse anti-CD68 (1:100 BioRad) were applied and allowed to incubate overnight at 4 °C. After washing with 1x PBS, slides were incubated with the following secondary antibodies for 2 hours at room temperature: Alexa Fluor 488 goat anti-mouse IgG (1:100, Abcam), and Alexa Fluor 594 goat anti-rabbit IgG (1:100, ThermoFisher). Then, the slides were washed three times with 1x PBS and mounted with ProLong® Gold Antifade Mountant with DAPI for nuclei staining (Molecular Probes, Eugene, OR). Images were acquired using a Nikon Eclipse Ti2 microscope and were analyzed using ImageJ software (Version 1.53e, NIH, USA). Cells were counted in 6 different areas that were randomly chosen from the region of interest and averaged to be used for statistical analysis. For brain samples, the peri-lesional area was defined as being within 250–500 μm from the impact site. All counting was done while blinded to group identity.

#### In situ detection of DNA fragmentation by Tdt dUTP nicked end labelling (TUNEL).

The Click‐iT Plus TUNEL assay (C10617; Invitrogen) kit was used for the detection of *in situ* apoptosis in the spleen tissue (*n* = 6 per experimental group), according to the manufacturer’s protocol. Paraffin-embedded spleen tissue was sliced into 8 µm sections using a microtome and used for staining. The fluorescence signal was detected using an Eclipse Ti2 Nikon microscope, and the degree of apoptosis was quantified using QuPath software by calculating the average area of TUNEL+ cells relative to the white pulp in 5 different randomly chosen areas.

### Barnes maze test

Spatial learning and memory were tested using the Barnes maze (*n* = 7–10 per experimental group), as previously described [12]. Rats were habituated to the maze by doing two trials during which the rats were guided to the correct escape hole during the first trial and to the incorrect then correct hole during the second trial. In both trials, the rats were allowed to stay in the escape box for 2 minutes. The acquisition phase included 4 trials for four consecutive days (again, prior to surgery). The rats were placed underneath a start semi-opaque tube located in the center of the maze. The trial began by removing the tube and allowing the rats to freely explore and search for the escape hole for 180 seconds or until the rat found the escape box. If the rat failed to locate the escape box, it was gently guided to the hole by the experimenter. For each trial, the latency to enter the escape box for each rat was recorded. Both the habituation and acquisition phases were performed prior to the surgery day. The probe trial was performed on PID 1 and included one 60-second run for each rat with the escape hole replaced with a dummy hole. During all trials, rats’ activity was recorded through an overhead camera connected to Smart tracking software (Panlab Harvard Apparatus, Holliston, MA), and videos were used to analyze data. To eliminate odor cues, the maze and escape box were cleaned with 70% ethanol after each trial.

### Statistical analysis

Data were analyzed using Prism version 10.1.0 (GraphPad Software, San Diego, CA). Graphical data are presented with individual data points as mean ± standard error of the mean (SEM) and compared by one-way Analysis of variance (ANOVA) followed by Tukey’s post-hoc test to perform multiple inter-group comparisons. Comparisons are reported with the overall F-value of the ANOVA, and the corresponding *p*-value with post-hoc comparisons were performed only if the overall *p*-value was statistically significant (*p* < 0.05). Data were evaluated for outliers using the Robust regression and Outlier removal (ROUT) method, and no outliers were noted [21].

## Results

### Bacterial load in the lung following bacterial inoculation

**Fig 2:**
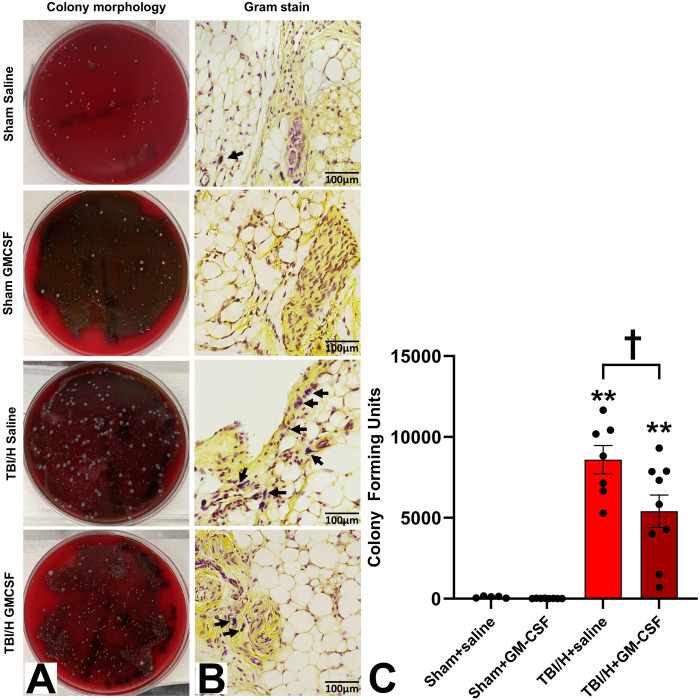
CFU counts and Gram stain of lung tissues 2 days following the injury. (A) Lung tissue from all groups was plated on blood agar and incubated for 24 hours for bacterial colony count. (B) Lung tissue sections (100x) were stained with the standard Gran stain to assess the presence of *Streptococcus pneumoniae* bacteria. Black arrows indicate gram positive bacteria. (C) Analysis of the bacterial colony count showed a significant increase in the TBI/H+saline and TBI/ + GM-CSF, as compared to either Sham group (** p < 0.001); n = 7-9 per experimental group. There was a significant decrease in the TBI/H + GM-CSF group in comparison to TBI/H+saline group (†p < 0.05). Abbreviations include: granulocyte-macrophage colony-stimulating factor (GM-CSF), and traumatic brain injury plus hemorrhage (TBI/H).

We quantified the effect of GM-CSF treatment on lung bacterial colonization following intranasal inoculation with *S. pneumoniae*. Lung tissue homogenates were plated on blood agar plates, and colonies were counted as CFU/mL sample (Fig 2A, F3,25 = 29.41, p < 0.0001). We used Gram staining to confirm the presence of Gram-positive bacteria (Fig 2B). For the sham groups, there was a low number of CFUs with no significant intergroup differences. Both injury groups showed significant increases in CFUs, as compared to the sham groups (*p* < 0.001 for all intergroup comparisons between the injured and sham groups). When comparing the injured groups (i.e., TBI/H+saline with TBI/H + GM-CSF), there was a significant reduction in the pulmonary CFUs in animals treated with GM-CSF (*p* = 0.023).

### Innate immune function in whole blood and spleen

**Fig 3:**
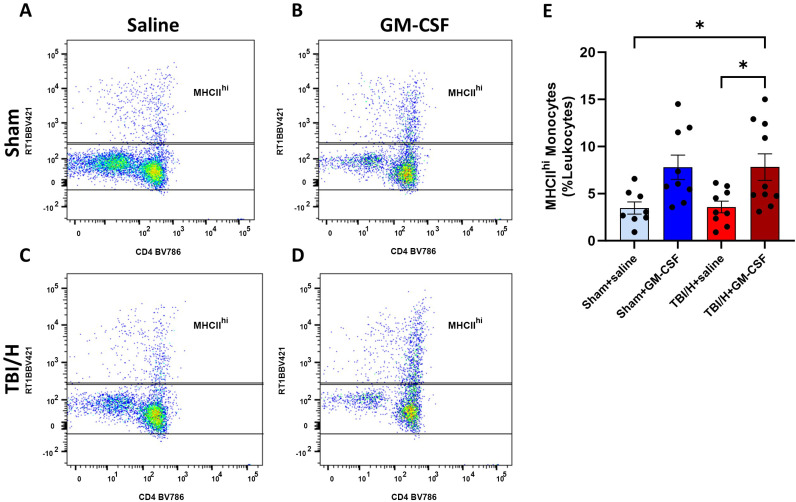
Measurement of circulating MHCII^hi^ monocytes using flow cytometry. MHCII^hi^ monocytes in whole blood were compared in all groups 1 day following the injury. *Post hoc* analysis showed a significant increase in the MHCII^hi^ monocyte cells in the GM-CSF groups, as compared to their relative controls (* p < 0.05); n = 8–10 per experimental group. Abbreviations include: granulocyte-macrophage colony-stimulating factor (GM-CSF), and traumatic brain injury plus hemorrhage (TBI/H).

To examine the effect of GM-CSF on inducing the innate immune response following sham or TBI/H injury, we assessed class II major histocompatibility complex (MHCII) expression by flow cytometry as an indicator of antigen-presenting capacity of circulating blood monocytes (F3,32 = 4.987, p = 0.006). Monocytes expressing elevated levels of MHCII molecules (MHCII^hi^) were assessed as a percentage of leukocytes. On PID 1 (Fig 3), we found a significant increase in the percentage of MHCII^hi^ expressing monocytes in the TBI/H + GM-CSF, as compared with Sham+saline or TBI/H+saline (*p* = 0.044 and 0.042, respectively). Comparison of mean expression of MHCII^hi^ between Sham+saline and Sham+GM-CSF groups did not meet statistical significance (*p* = 0.053).

**Fig 4:**
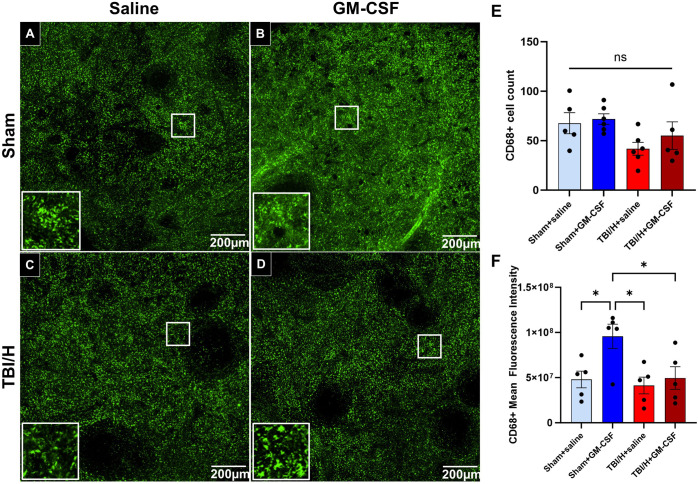
Immunofluorescence expression of CD68 marker in the spleen. (A–D) Representative fluorescence images of CD68 + cells in the spleen sections of the four cohorts were captured at 40x magnification (inset pictures are 600x). (E) Analysis of CD68 + cell count showed non-significant changes between groups. (F) Analysis of mean fluorescence intensity showed a significant increase in the sham+GM-CSF group, as compared to the sham+saline and TBI/H saline groups (* p < 0.05); n = 5–6 per experimental group. Abbreviations include: granulocyte-macrophage colony-stimulating factor (GM-CSF), and traumatic brain injury plus hemorrhage (TBI/H).

We also examined splenic tissue-resident macrophages by immunohistochemical labeling of CD68 (Fig 4A–D) with measurement of two parameters; CD68 + cell counts and mean fluorescence intensity (F3,18 = 2.34, p = 0.10 and F3,16 = 4.92, p = 0.013, respectively). Analysis of CD68 + cell counts revealed no significant differences in inter-group comparison (*p* = 0.11, Fig 4E); however, the mean fluorescence intensity (*p* = 0.013, Fig 4F) of the CD68 + cells in the Sham+GM-CSF group was higher, as compared to all other groups (p ≤ 0.046).

### T cell presence in the spleen

**Fig 5:**
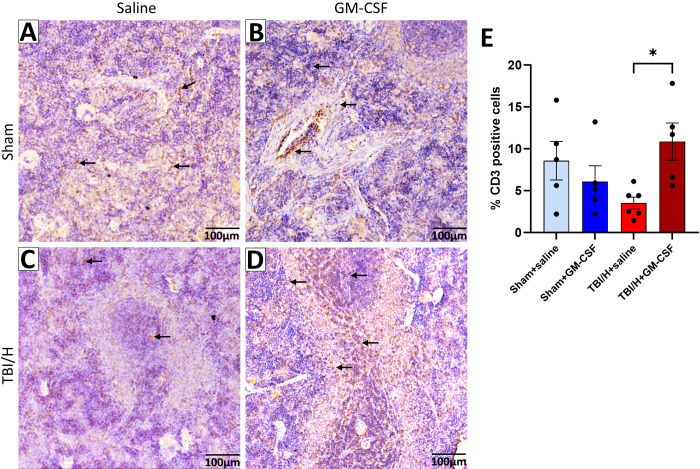
Immunohistochemical expression of CD3 marker in the spleen. (A–D) Representative images of CD3 + cells in the spleen sections of the four cohorts were captured at 200x magnification (inset pictures are 600x). (E) Analysis of the percent area of CD3 + cells showed a significant increase in the TBI/H + GM-CSF, as compared to the TBI/H+saline group. Black arrows indicate CD3 + cells (* p < 0.05); n = 5-8 per experimental group. Abbreviations include: granulocyte-macrophage colony-stimulating factor (GM-CSF), and traumatic brain injury plus hemorrhage (TBI/H).

To further investigate the adaptive immune response in the spleen we evaluated the expression of CD3, a marker of mature T cell lymphocytes, by immunohistochemistry (Fig 5A–D, F3,17 = 3.2, p = 0.048). Our results demonstrate that the expression of CD3 + T cells in the sham groups was equivalent (Fig 5E). The number of T-cells was significantly lower in the TBI/H+saline group, as compared to the TBI/H + GM-CSF group (Fig 5E, p = 0.041).

### Splenocyte apoptosis in the white pulp

**Fig 6:**
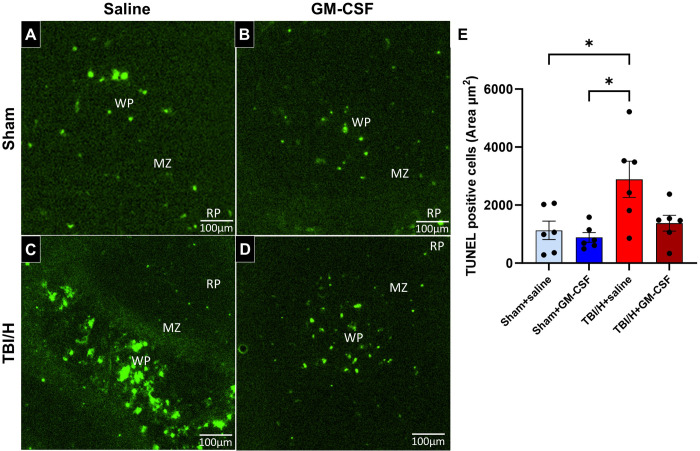
Assessment of DNA fragmentation (TUNEL staining) in spleen tissue following injury. (A–D) Representative fluorescence images of TUNEL-stained spleen sections showing the area of TUNEL-positive cells in the white pulp (WP) region (400x). (E) Fluorescence analysis showed a significant increase in the white pulp’s TUNEL-positive areas in the TBI/H+saline group, as compared to all groups (* p < 0.05); n = 6 per experimental group. Abbreviations include: granulocyte-macrophage colony-stimulating factor (GM-CSF), marginal zone (MZ), red pulp (RP), and traumatic brain injury plus hemorrhage (TBI/H).

A TUNEL assay was used to test for the degree of splenic apoptosis (in the white pulp) (Fig 6, F3,20 = 5.49, p = 0.0064). The TUNEL assay revealed that there was a significant increase in TUNEL+ cells in the TBI/H+saline group within the white pulp, as compared with Sham+saline or Sham+GM-CSF (*p* = 0.02 and 0.0075, respectively). When comparing TBI/H + GM-CSF with the sham groups, no significant difference was noted (*p* > 0.80).

### Neuroinflammatory response in the brain

**Fig 7:**
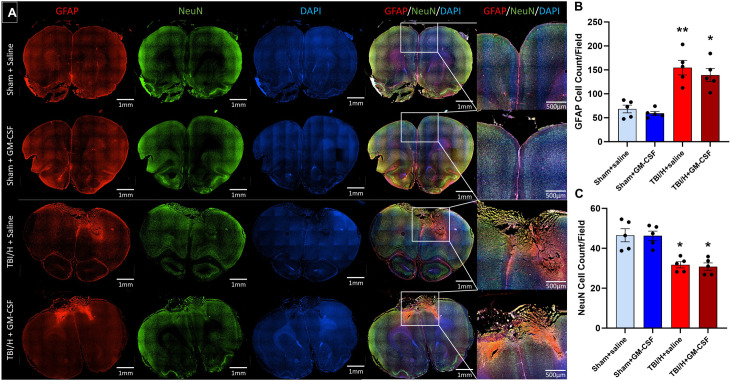
Immunofluorescence expression of astrocyte and neuronal cells in brain tissues following the injury. Astrocytes activation (red) and neuronal cells (green) were evaluated by immunohistochemical staining with GFAP and NeuN antibodies respectively, and nuclei were identified with DAPI (blue). (A) Whole brain representative fluorescence images were created by capturing and stitching multiple images at 100x magnification (inset pictures were zoomed in to show the lesion area). (B) Analysis of astrocyte cell counts in the peri-lesional areas showed a significant increase in cell numbers in the TBI/H groups, as compared to the Sham + saline group (* p < 0.05, ** p < 0.001). (C) Analysis of NeuN+ positive cells in the peri-lesional areas showed a significant decrease in the neuronal cell numbers in the TBI/H groups, as compared to the Sham + saline group (* p < 0.05); n = 5–6 per experimental group. Abbreviations include: 4’,6-diamidino-2-phenylindole (DAPI), glial fibrillary acidic protein (GFAP), granulocyte-macrophage colony-stimulating factor (GM-CSF), neuronal nuclear protein (NeuN), and traumatic brain injury plus hemorrhage (TBI/H).

To quantify the impact of GM-CSF treatment on neuroinflammation in the brain, we investigated the level of glial cell activation and neuronal loss in the peri-lesional area two days following the injury. Glial fibrillary acidic protein (GFAP), a marker for astrocytes was used to assess astrocytes (Fig 7, F3,16 = 18.9, p < 0.0001). The numbers of GFAP+ cells were comparable in the sham groups with no statistical differences (Fig 7B, *p* = 0.94,) noted. However, as compared to either Sham+saline or Sham+GM-CSF, there was a significant increase in the total cell counts for GFAP+ cells in the TBI/H+saline samples (*p* < 0.001) and in the TBI/H + GM-CSF samples (*p* = 0.002 and *p* < 0.001, respectively). There was no significant difference in number of GFAP+ cells when comparing TBI/H+saline and TBI/H + GM-CSF (*p* = 0.76).

Additionally, we performed immunofluorescence staining of brain sections with NeuN antibody, a biomarker for neurons, to assess neuronal density in the peri-lesional region (Fig 7C, F3,16 = 13.91, p = 0.0001). As expected, TBI/H with or without GM-CSF caused a significant reduction of peri-lesional NeuN+ cells in the TBI/H groups, as compared to the sham groups (p < 0.002, for all comparisons between sham groups and TBI/H groups). There was no difference in the NeuN+ cell counts between the TBI/H groups or between the sham groups.

**Fig 8:**
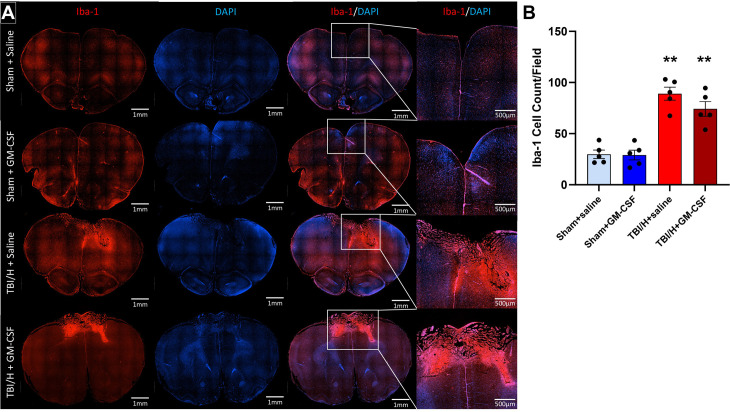
Immunofluorescence expression of microglial cells in brain tissues following the injury. Microglia activation (red) was evaluated by immunohistochemical staining with Iba-1 antibody and nuclei were identified with DAPI (blue). (A) Whole brain representative fluorescence images were created by capturing and stitching multiple images at 100x magnification (inset pictures were zoomed in to show the lesion area). (B) Analysis of microglial cell counts in the pericontusional areas showed a significant increase in cell numbers in the TBI/H groups, as compared to the sham groups (** p < 0.001); n = 5-6 per experimental group. Abbreviations include: 4’,6-diamidino-2-phenylindole (DAPI), granulocyte-macrophage colony-stimulating factor (GM-CSF), ionized calcium-binding adapter molecule 1 (Iba-1), neuronal nuclear protein (NeuN), and traumatic brain injury plus hemorrhage (TBI/H).

Microglia were also quantified (Fig 8A) using ionized Ca^2+^-binding adapter molecule 1 (Iba-1), a microglia marker (Fig 8B, F3,16 = 28.72, p < 0.0001). No significant difference was noted when comparing Iba-1 + cell counts between the Sham+saline group and the Sham+GM-CSF group (p = 0.99). As compared to either Sham+saline or Sham+GM-CSF, there was a significant increase in the total cell counts for Iba-1 + cells in the TBI/H+saline samples (*p* < 0.001 for both comparisons) and in the TBI/H + GM-CSF samples (*p* < 0.001 for both comparisons). There was no significant difference noted when comparing Iba-1 + cells/field between TBI/H+saline and TBI/H + GM-CSF groups (*p* = 0.29).

### Neurobehavioral testing

**Fig 9:**
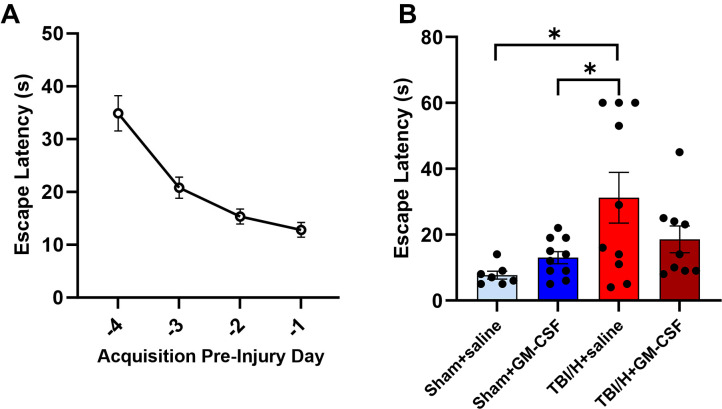
Barnes maze testing in animals on post-injury day 1. The behavioral testing protocol is as outlined in Fig 1. (A) Briefly, animals received acclimation and then four days of acquisition with expected decreasing mean times to escape. (B) On post-injury day 1, animals received a probe test, and escape latencies for each group are shown. There was a significant difference noted between TBI/H+saline and both Sham+saline and Sham+GM-CSF (*p < 0.05); n = 7–10 per experimental group. Abbreviations include: granulocyte-macrophage colony-stimulating factor (GM-CSF), second (s), and traumatic brain injury plus hemorrhage (TBI/H).

To determine the impact of the injury/infection model and GM-CSF treatment on neurological function, Barnes maze testing was performed to assess spatial memory. All animals received Barnes maze acclimation and four days of acquisition prior to injury/infection and treatment. Animals demonstrated a typical pattern of decreasing time for escape latency (Fig 9A, F3,32 = 4.23, p = 0.0125). A probe trial was conducted on PID 1. Analysis of the probe trial data showed no significant differences between the sham groups; however, there was a significant increase in escape latency in the TBI/H+saline, as compared to the Sham+saline or the Sham+GM-CSF groups (*p* = 0.013 and 0.042, respectively, Fig 9B). In contrast, no significant difference was seen in escape latency when comparing TBI/H + GM-CSF to either sham treatment group (Fig 9B).

## Discussion

Our results demonstrate that systemic post-injury treatment with GM-CSF is associated with improved host defense in our juvenile rat TBI/H model as evidenced by reduced bacterial load following pneumonia challenge; increased percentage of blood monocytes expressing elevated levels of MHC II molecules; and preservation of splenic lymphocytes. Post-injury treatment with GM-CSF was *not* associated with increased astrocyte or glial proliferation at the perilesional site; increased neuronal loss; or impairment of spatial memory. In fact, post-injury treatment with GM-CSF was associated with spatial memory performance that was no different from sham injured animals.

While we have previously shown that treatment with GM-CSF can reverse TBI/H-induced systemic immunosuppression, the presented experiments represent the first evidence that treatment with GM-CSF can reduce lung bacterial load in an experimental model of nosocomial infection after traumatic injury. This is clinically relevant as severe TBI with an extracranial injury has been associated with an increased risk of nosocomial infections in children [6]. We chose the one and two-day post-injury time point, as this timing coincides with the maximal decline in immune function noted in our injury model [12,13,18].

One of the major consequences following polytrauma or severe neurotrauma is the increased risk of lung infections, and a resultant increase in mortality risk [22,23]. TBI patients frequently present with extracranial injuries which can worsen the neurological outcomes and further increase the risk of nosocomial infections [24,25]. Based on both our prior work and the work of others, TBI-induced systemic immune suppression, including impairment of both innate and adaptive immunity, represents a major mechanism in the development of such infections [8,26]. This post-injury immune suppression includes impaired monocyte phagocytic activity and reduced cytokine production capacity in circulating leukocytes [10]. In agreement with this, we have previously shown reduced ex-vivo lipopolysaccharide-induced TNFα production in whole blood following TBI/H injury in our model [12]. GM-CSF is a T_H_1 cytokine that not only stimulates the generation and proliferation of granulocytes and macrophages from the bone marrow, but is also known to improve responsiveness and function of existing innate immune cells [15]. We show that post-injury GM-CSF treatment resulted in an increased percentage of blood monocytes expressing elevated levels of MHC II molecules. Furthermore, the differential response between sham and TBI/H animals receiving GM-CSF may be due to the underlying injury and resultant inflammation, which could alter the immune response when comparing injured and uninjured animals [27].

Although lymphocytes are not typically the direct target of GM-CSF [28], adaptive immune cells such as T-cells and B-cells can be indirectly modulated by GM-CSF through its action on antigen presenting cells (APC), including their release of IL-23 and IL-1β [29,30]. GM-CSF is known to be important for the activation and priming of T-cells and for optimizing interactions between APCs and T-cells [31,32]. Similarly, an earlier study reported an improved T helper cell response following treatment with GM-CSF in mouse spleen tissue [33]. Moreover, there was an increase in spleen cellularity in response to GM-CSF treatment [33]. In line with this evidence, we found that GM-CSF treatment preserved the number of CD3 + T-cells in the spleen of TBI/H animals; however, the different T-cell phenotypes that were affected by GM-CSF are yet to be studied.

Additionally, our data showed that while TBI/H alone was associated with significantly increased splenic apoptosis (as compared with uninjured animals), spleens from TBI/H animals treated with GM-CSF showed no significant difference from uninjured animals. Splenocyte apoptosis has been shown to occur in adult and pediatric sepsis non-survivors [34], and we have seen similar findings in prior work with our TBI/H animal model [13]. GM-CSF has been shown in previous studies to promote cell survival through multiple pathways including phosphatidylinositol-3-kinase (PI3K) [35] and JAK/STAT5-Bcl-2 [36] and induces cell proliferation through Erk and NF-kB signaling [37,38].

Neuroinflammatory reaction is a major hallmark of TBI that mediates TBI pathology and other neurodegenerative processes [39]. Accordingly, there is a valid concern that treatment with an immune stimulant such as GM-CSF could aggravate the neuroinflammatory response in the brain and exacerbate secondary cell death following TBI. To address this concern, we evaluated glial cell activation and neuronal loss in the peri-lesional area following injury. Interestingly, we did not find any significant difference in microglial, astrocyte, and neuronal cell counts in the GM-CSF treated groups when comparing these animals to their sham or injured counterparts. This is in agreement with previous studies that showed a neuroprotective effect of GM-CSF in animal models of Parkinson’s disease [40], Alzheimer’s disease [41], stroke [42], and TBI [43,44]. Likewise, treatment with GM-CSF did not lead to an exacerbation of the spatial memory deficit seen on Barnes maze testing following TBI/H. Our present findings agree with our prior research using the polytraumatic TBI model where 7 days of daily dosing of GM-CSF was associated with improved leukocyte response to immunogen without an increased functional deficit or increases in pro-inflammatory cytokines or activated microglia in the lesional or peri-lesional areas [18].

To our knowledge, this is the first preclinical study to test the therapeutic effect of GM-CSF in mitigating respiratory infection caused by *S. pneumoniae* in a juvenile model of TBI/H. Both clinical and preclinical studies have reported cellular immunosuppression following TBI injury and increased susceptibility to infections [8,45]. Perhaps the most clinically relevant finding in our present study is the reduction of lung bacterial load in the GM-CSF treated TBI/H animals, providing evidence of functionally preserved host defense. This is potentially explained through the preservation of systemic antigen-presenting capacity. This is important because studies have shown that it is the monocyte and macrophage lineage that is responsible for early immune responses including bacterial clearance [46]. We chose *S. pneumoniae* because it is a common pathogen in pneumoniae in critically ill patients [47] and has been previously used in pneumonia models in Sprague Dawley rats [48].

Our study has several limitations. We only investigated the effect of GM-CSF during the early acute (2 days) phase following the injury; however, various immune-related pathways are activated during the late chronic phase which can affect TBI pathology. Longer-term studies with GM-CSF treatment may be warranted. Secondly, understanding the different subphenotypes of lymphocyte cells (T-cells in particular) that are modulated by GM-CSF treatment is important to better understand the mechanisms by which GM-CSF works in this setting. This would also help in identifying therapeutic target cells/markers that could be studied further. Although we observed reduced bacterial load in the lung tissue, we did not measure the degree of functional impairment of the lungs prior to sacrifice. As this experiment was designed to be proof of concept, to determine that GM-CSF treatment could reduce lung bacterial load, our inoculum did not lead to pneumonitis or bacteremia. Future studies will be conducted with sufficient inoculum to lead to systemic infection. Another limitation of our study is that we did not specifically address the effect of GM-CSF on platelets and megakaryocytes. GM-CSF can have variable effects on platelet count [49], and this interaction could potentially impact the hemostatic balance and recovery process in TBI models with hemorrhage. Future research should explore this aspect for a more comprehensive understanding. Finally, our model, while generating significant neurologic and systemic injury, does not fully recapitulate the degree of injury seen in injured children who are managed with intensive care.

## Conclusion

In this study, we described the role of GM-CSF in preventing immunosuppression and attenuating respiratory infection following a model of TBI/H injury and bacterial inoculation. Our data suggest that GM-CSF enhanced the percentage of blood monocytes with elevated MHCII expression and preserved splenic lymphocyte populations. We also demonstrated that GM-CSF treatment does not aggravate brain neuroinflammatory responses or impair spatial memory. This study highlights the potential of GM-CSF as a therapeutic option to reduce the risk of nosocomial infection following pediatric polytrauma which should be the subject of further study.

## Supporting information

S1 Fig At post-natal day 28, rats received either the TBI/H injury model or sham injury. Following injury, rats were inoculated by intra-nasal application of *S. pneumoniae*. Rats were then sacrificed at either 2 or 4 days post-injury/inoculation, and lungs were harvested and then plated on blood agar. Colony forming units (CFU) are shown. A significant increase in CFU is noted at post-injury day 2 when comparing TBI/H treated animals with sham (** p < 0.001); n ≥ 3 per experimental group.
(TIF)

S2 Fig The flow cytometry gating protocol used for separation of MHCII^hi^ cells is shown.
(TIF)

S1 Data File Data file. (CSV)
