# Supplementary figures and images for: Granulocyte-macrophage colony-stimulating factor reduces lung bacterial load following traumatic brain injury and hemorrhage polytrauma in a juvenile rat model
Source: PLoS One. 2025 May 19;20(5):e0323674. doi: 10.1371/journal.pone.0323674 (PMC12088021; doi:10.1371/journal.pone.0323674)

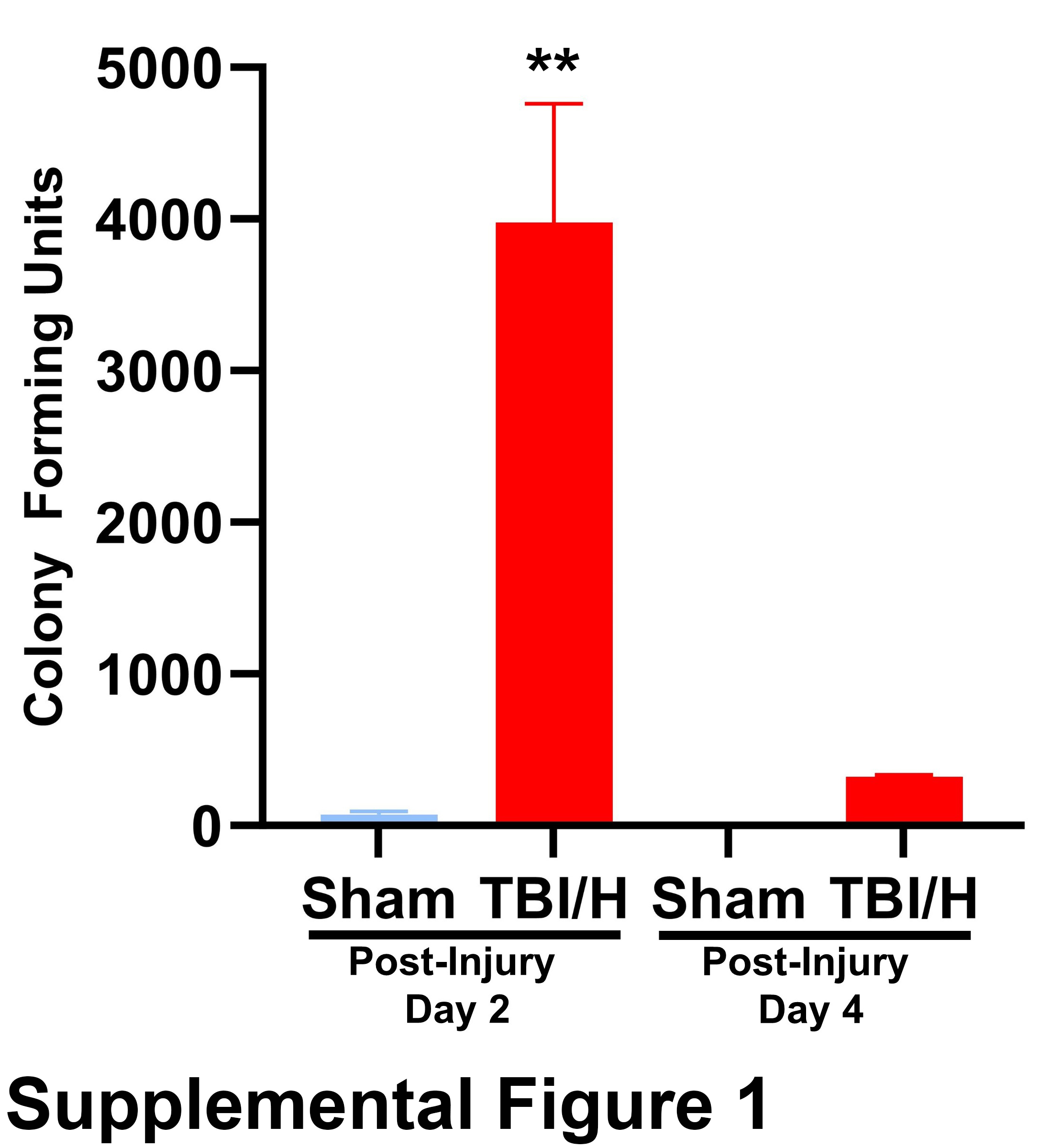

Supplement: S1 Fig — Following injury, rats were inoculated by intra-nasal application of S. pneumoniae. Rats were then sacrificed at either 2 or 4 days post-injury/inoculation, and lungs were harvested and then plated on blood agar. Colony forming units (CFU) are shown. A significant increase in CFU is noted at post-injury day 2 when comparing TBI/H treated animals with sham (** p < 0.001); n ≥ 3 per experimental group. (TIF) [file pone.0323674.s001.tif]

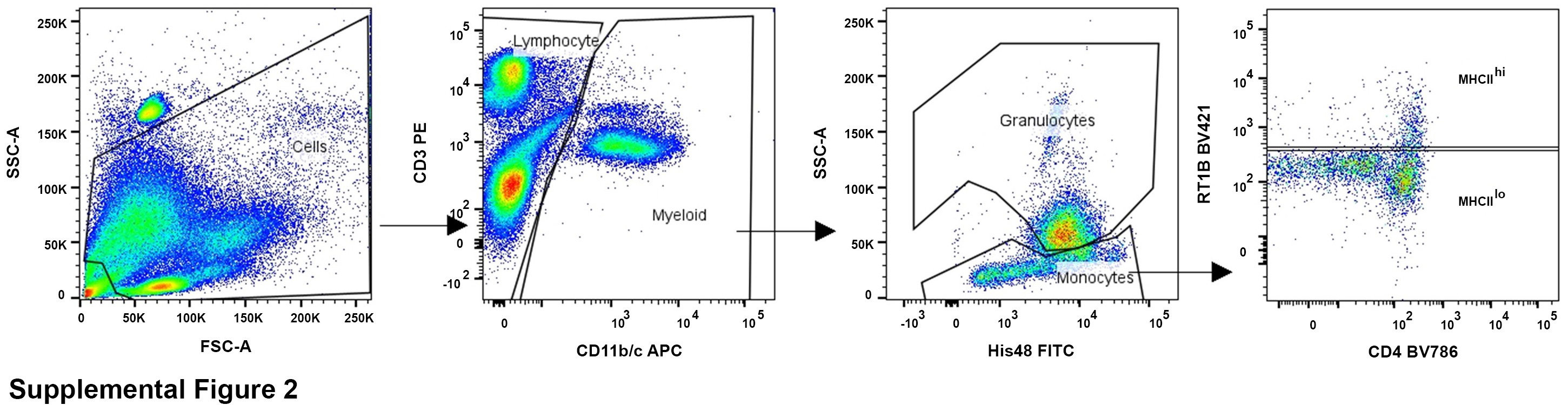

Supplement: S2 Fig — The flow cytometry gating protocol used for separation of MHCIIhi cells is shown. (TIF) [file pone.0323674.s002.tif]
